# White-Hat Worm to Fight Malware and Its Evaluation by Agent-Oriented Petri Nets †

**DOI:** 10.3390/s20020556

**Published:** 2020-01-19

**Authors:** Shingo Yamaguchi

**Affiliations:** Graduate School of Sciences and Technology for Innovation, Yamaguchi University, Ube 755-8611, Japan; shingo@yamaguchi-u.ac.jp

**Keywords:** IoT, cybersecurity, malware, DDoS, bot, botnet, Petri net

## Abstract

A new kind of malware called *Mirai* is spreading like wildfire. Mirai is characterized by targeting Internet of Things (IoT) devices. Since IoT devices are increasing explosively, it is not realistic to manage their vulnerability by human-wave tactics. This paper proposes a new approach that uses a white-hat worm to fight malware. The white-hat worm is an extension of an IoT worm called *Hajime* and introduces lifespan and secondary infectivity (the ability to infect a device infected by Mirai). The proposed white-hat worm was expressed as a formal model with agent-oriented Petri nets called *PN${}^{2}$*. The model enables us to simulate a battle between the white-hat worm and Mirai. The result of the simulation evaluation shows that (i) the lifespan successfully reduces the worm’s remaining if short; (ii) if the worm has low secondary infectivity, its effect depends on the lifespan; and (iii) if the worm has high secondary infectivity, it is effective without depending on the lifespan.

## 1. Introduction

In September 2016, unprecedented Distributed Denial-of-Service (DDoS) attacks knocked out Twitter, Amazon, and other major sites. They were launched by lots of Internet of Things (IoT) devices which were infected by a new kind of malware called *Mirai*. Mirai infects IoT devices one after another and makes them a botnet to perform DDoS attacks [1]. Mirai is spreading like wildfire and actually has infected over 300,000 IoT devices in 164 countries [2]. This so-called *Mirai pandemic* results from characteristics of IoT devices such as (i) increasing explosively, (ii) existing anywhere, and (iii) using easy-to-guess passwords. Thus, DDoS attacks brought by IoT devices tend to become massive and disruptive [3].

There are some mitigation methods against Mirai. One method proposed by US Computer Emergency Readiness Team (US-CERT) [4] is to reboot the device infected by Mirai. This is simple, but the device would be reinfected soon if it is not updated. A promising method is to use a kind of IoT worms called *Hajime* as a vaccine against Mirai. Hajime infects IoT devices one after another and blocks ports that Mirai uses to infect [5]. However, there are few quantitative evaluations of Hajime’s effect.

Since November 2017, the authors of Ref. [6] have evaluated Hajime’s effect quantitatively. They regarded the battle between Mirai and Hajime as a multi-agent system and expressed it with agent-oriented Petri net called *Petri Nets in a Petri Net* (PN${}^{2}$ for short) [7]. The evaluation result showed that Hajime protected IoT devices from Mirai’s infection. However, the devices became infected by Hajime instead. For now, unlike Mirai, Hajime does not have any DDoS capability. However, Hajime has a remote control mechanism, which is an unfavorable attribute.

IoT devices are increasing explosively. Thus, it is not realistic to manage their vulnerability against Mirai by human-wave tactics. In this paper, we propose a new approach that uses a white-hat worm to fight Mirai. We first extend Hajime to become the white-hat worm by introducing lifespan and secondary infectivity (the ability to infect a device infected by Mirai). Next, we construct a PN${}^{2}$ model representing the white-hat worm. Then, we evaluate the effect of the white-hat worm against Mirai through the simulation of the model.

The rest of this paper is organized as follows: Section 2 surveys the related work. Section 3 gives the design of the white-hat worm and its PN${}^{2}$ model. Section 4 presents the simulation for evaluating the effect of the white-hat worm against Mirai. Section 5 summarizes our key points and gives future work.

## 2. Related Work

### 2.1. Mirai and Hajime

Mirai is a kind of malware that infects IoT devices. It turns them into bots and creates a botnet. The botnet becomes a hotbed of large-scale DDoS attacks. In August 2016, Mirai was found by a malware research group *MalwareMustDie*. The first attack happened in September 2016. Since then, Mirai has been spreading like wildfire around the world.

Mirai takes the following two stages. The first is the infection stage. Mirai searches for an IoT device using port 23 or 2323. Once Mirai finds such a device, it tries to log-in with easy-to-guess passwords. If succeeding in log-in, Mirai downloads an architecture-dependent code from the Command and Control (C&C) server and executes it. As a result, the device becomes a bot. The second is the attack stage. Once an attacker issues a command, the C&C server delivers it to bots. All the bots begin a DDoS attack on the target specified by the attacker. In November 2016, US-CERT announced that Mirai can be removed if the infected device is rebooted. For the detail of Mirai, refer to Ref. [8].

There are some systems that detect IoT malware like Mirai. Bezerra et al. [9] proposed a host-based malware detection system, named IoTDS (Internet of Things Detection System). It analyzes the host’s CPU and memory utilization, CPU temperature, and the number of running tasks and classifies its behavior as malicious or legitimate. On one hand, with the continuous development of machine learning algorithms, some learning-based malware detection systems have been proposed. However, these learning-based detection systems are often vulnerable to adversarial samples. Liu et al. [10] proposed an automated testing framework that can generate an effective adversarial sample without affecting the features of the IoT application. This framework enables us to perform black-box testing. However, IoTDS and the learning-based malware detection systems detect malware but don’t take any action for the detected malware themselves. Ceron et al. [11] proposed a network layer that adapts itself to mitigate the network traffic generated by IoT malware. It can modify the traffic at the network layer based on the actions performed by the malware. However, the network layer is not something to remove the malware.

Hajime is a kind of worms that infects IoT devices. In October 2016, Hajime was found by the security research group at Rapidly Networks, Inc. [5]. Hajime aims at creating a botnet like Mirai. However, there is a crucial difference between Mirai and Hajime. Hajime has no capability for DDoS attacks [12]. On the contrary, Hajime blocks the ports that Mirai accesses to infect the device and displays a warning message to inform the user of the risk of malware. Hajime is an unethical way but can be regarded as one of the mitigation methods against Mirai.

When utilizing worms like Hajime as a mitigation method against Mirai, there are several challenges to be overcome about viability, capability, legality, and ethics of the worms. Molesky  et al. [13] discussed some of the challenges from the viewpoints of individual, business and government. They proposed a perspective for utilizing worms through balancing them. However, they provided no more than qualitative concepts and have not evaluated them quantitatively.

### 2.2. PN${}^{2}$ and Modeling

Yamaguchi et al. [14] regarded the battle between Mirai and Hajime as a multi-agent system and expressed it with PN${}^{2}$.

A PN${}^{2}$ is intuitively a Petri net (called as *environment net*) whose tokens are again Petri nets (called as *agent nets*). Each agent net represents the state-transition of an agent, and the environment net specifies the interaction between agents. The number of tokens in the environment net may increase or decrease. This means the creation or deletion of agents. The transitions of two or more agent nets may fire simultaneously. This means an interaction between the corresponding agents. The combination of agents related to the interaction is dynamically decided because PN${}^{2}$ allows dynamic bindings of transitions. Nakahori et al. [15] developed a tool, called as *PN2Simulator*, to edit and simulate PN${}^{2}$. For the detail of Petri nets and PN${}^{2}$, refer to Refs. [7,16].

There are some agent-oriented approaches in the research area in cybersecurity. For example, García-Magariño et al. [17] proposed a process called *PEABS* for constructing efficient multi-agent simulators. They applied it to some cybersecurity issues and successfully developed simulators like ABS-SecurityUAV [18]. Such previous research is oriented to the expressiveness of agent-based systems. In contrast, our approach based on PN${}^{2}$ is oriented to mathematical analysis. The semantics of PN${}^{2}$ enables us to formally describe the phenomenon and to analyze with Petri net’s properties and their abundant analysis methods.

**Figure 1:**
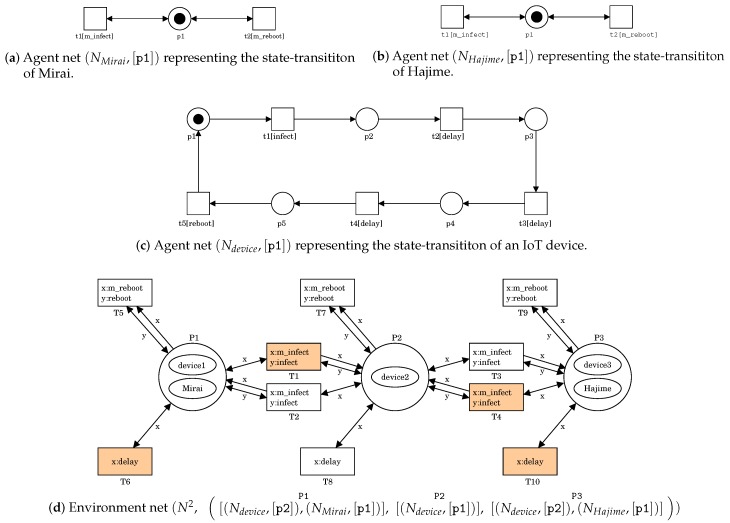
A PN${}^{2}$ model representing a battle between Mirai and Hajime.

PN${}^{2}$ enables us to model a battle between Mirai and Hajime. An example is shown in Figure 1. The agent net $N_{Mirai}$ of Figure 1a represents the state-transition of Mirai. Transition t1 (drawn by □) labeled as m_infect represents an infection action. Transition t2 labeled as m_reboot represents a reboot action. Place p1 forms a self-loop together with each transition. p1 possesses a token (drawn by •). A token distribution is called as a state and is denoted by a multi-set over the set *P* of places, i.e., $\left[p^{M(p)}|p\in P,M\left(p\right)>0\right]$, where M(p) is the number of tokens at *p*. $N_{Mirai}$ with state [p1] being denoted by $\left(N_{Mirai},\left[p1\right]\right)$. Since p1 has a token, the transitions can fire repeatedly. This means that Mirai repeatedly infects a device and is deleted by a reboot action.

The agent net $N_{Hajime}$ of Figure 1b represents the state-transition of Hajime. It is the same net structure as Mirai because Hajime has the same capabilities except for the DDoS one.

The agent net $N_{device}$ of Figure 1c represents the state-transition of an IoT device. Transition t1 labeled as infect represents an infection action. Even if it becomes a bot, it can be returned to normal by rebooting. However, it is not always rebooted immediately after the infection. Thus, we should introduce some delay until the reboot. Transitions t2, t3, and t4 labeled as delay respectively represent a delay action. Transition t5 labeled as reboot represents a reboot action.

The environment net $N^{2}$ of Figure 1d represents an IoT network of linear topology which consists of three nodes. The first node connects with the second node and the second node connects with the third node. Each node is expressed as a place. A token (drawn by ⬭) represents an IoT device, Mirai, or Hajime. We assume in this paper that each node has one device. Place P1 possesses two tokens representing Mirai and a device device1. This means that Mirai infects device1. Place P2 possesses only one token representing a device device2. This means that device2 is normal. Place P3 possesses two tokens representing Hajime and a device device3. This means that Hajime infects device3. A transition represents an interaction among them, e.g., an infection of Mirai to a device. PN2Simulator can highlight which transition is firable in red. For each place *p* in $N^{2}$, let s(p) denote a multi-set of agent nets corresponding to tokens at *p*. Any state of $N^{2}$ is denoted by a vector of all s(p)’s. The state of Figure 1d is written as follows:

$$\begin{array}{c} s_{0}=(\overset{P1}{[\left(N_{device},\left[p2\right]\right),\left(N_{Mirai},\left[p1\right]\right)],}\;\overset{p2}{[\left(N_{device},\left[p1\right]\right)],}\;\overset{p3}{\left[\left(N_{device},\left[p2\right]\right),\left(N_{Hajime},\left[p1\right]\right)\right]}). \end{array}$$

### 2.3. Simulation Evaluation

**Figure 2:**
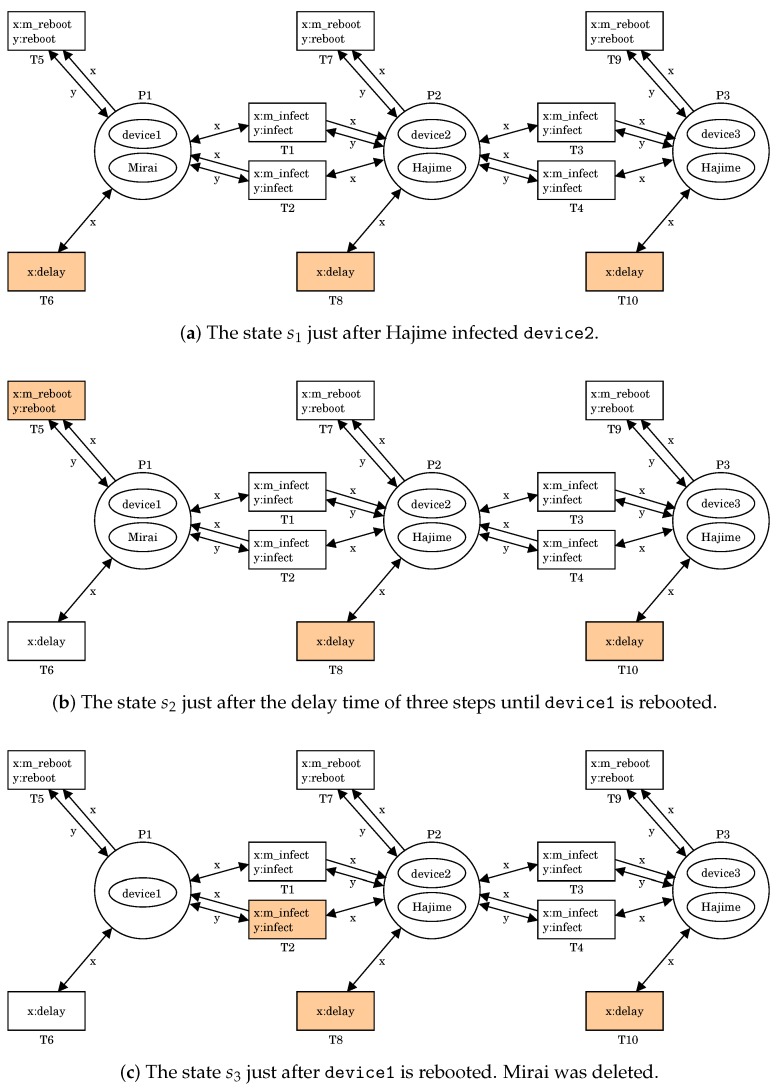
An execution of $\left(N^{2},s_{0}\right)$.

We can simulate the battle between Mirai and Hajime by using the PN${}^{2}$ model given in Section 2.2. Figure 2 shows an execution of $\left(N^{2},s_{0}\right)$. Note that $\left(N^{2},s_{0}\right)$ is shown in Figure 1d. In $s_{0}$, there are four firable transitions T1, T4, T6, and T10 because

- For T1, x:m_infect and y:infect can be respectively bounded with t1 in $\left(N_{Mirai},\left[p1\right]\right)$ at P1 and t1 in $\left(N_{device},\left[p1\right]\right)$ at P2.
- For T4, x:m_infect and y:infect can be respectively bounded with t1 in $\left(N_{Hajime},\left[p1\right]\right)$ at P3 and t1 in $\left(N_{device},\left[p1\right]\right)$ at P2.
- For T6, x:delay can be bounded with t2 in $\left(N_{device},\left[p2\right]\right)$ at P1.
- For T10, x:delay can be bounded with t2 in $\left(N_{device},\left[p2\right]\right)$ at P3.

Let us fire T4. It means that Hajime infects device2, i.e., Hajime at P3 produces a copy of itself into P2, and the copy infects device2. This results in a new state $s_{1}=$

$$\begin{array}{c} \left(\overset{P1}{[\left(N_{device},\left[p2\right]\right),\left(N_{Mirai},\left[p1\right]\right)],}\;\overset{P2}{[\left(N_{device},\left[p2\right]\right),\left(N_{Hajime},\left[p1\right]\right)],}\;\overset{P3}{\left[\left(N_{device},\left[p2\right]\right),\left(N_{Hajime},\left[p1\right]\right)\right]}\right) \end{array}$$

shown in Figure 2a.

In $s_{1}$, there are three firable transitions T6, T8, and T10 because, for each transition, x:delay can be bounded with t2 in $\left(N_{device},\left[p2\right]\right)$. T1 is no longer firable in $s_{1}$. This means that Hajime at P2 protects device2 from Mirai. Let us fire T6 three times. It means that the delay time of three steps passed. This results in a new state $s_{2}=$

$$\begin{array}{c} \left(\overset{P1}{[\left(N_{device},\left[p5\right]\right),\left(N_{Mirai},\left[p1\right]\right)],}\;\overset{P2}{[\left(N_{device},\left[p2\right]\right),\left(N_{Hajime},\left[p1\right]\right)],}\;\overset{P3}{\left[\left(N_{device},\left[p2\right]\right),\left(N_{Hajime},\left[p1\right]\right)\right]}\right) \end{array}$$

shown in Figure 2b.

In $s_{2}$, T5 becomes firable because x:m_reboot and y:reboot can be respectively bounded with t2 in $\left(N_{Mirai},\left[p1\right]\right)$ at P1 and t5 in $\left(N_{device},\left[p5\right]\right)$ at P1. Let us fire T5. It means a reboot of device1, i.e., Mirai at P1 is deleted and device1 gets back to normal. This results in a new state $s_{3}=$

$$\begin{array}{c} \left(\overset{P1}{[\left(N_{device},\left[p1\right]\right)],}\;\overset{P2}{[\left(N_{device},\left[p2\right]\right),\left(N_{Hajime},\left[p1\right]\right)],}\;\overset{P3}{\left[\left(N_{device},\left[p2\right]\right),\left(N_{Hajime},\left[p1\right]\right)\right]}\right) \end{array}$$

shown in Figure 2c.

Using the PN${}^{2}$ model, Yamaguchi et al. [14,19] have evaluated the effect of Hajime against Mirai. They used the PN${}^{2}$ model representing a lattice-structured network. The network consists of 25 (=5×5) nodes and each node has one device. They measured Mirai’s infection rate $R_{Mirai}$ after 1000 steps. $R_{Mirai}$ is given by

(1) $$R_{Mirai}=\frac{{\#}_{Mirai}}{{\#}_{device}},$$

where ${\#}_{device}$ is the number of devices and ${\#}_{Mirai}$ is the number of devices infected by Mirai. The parameters are as follows:

- The delay time δ until rebooting = 0, 1, 2, 3, or 4 steps.
- The initial number ${\#}_{Mirai}^{init}$ of devices infected by Mirai = 1.
- The initial number ${\#}_{Hajime}^{init}$ of devices infected by Hajime = 0, 1, 2, or 3.

Mirai and/or Hajime were initially put at random nodes.

**Figure 3:**
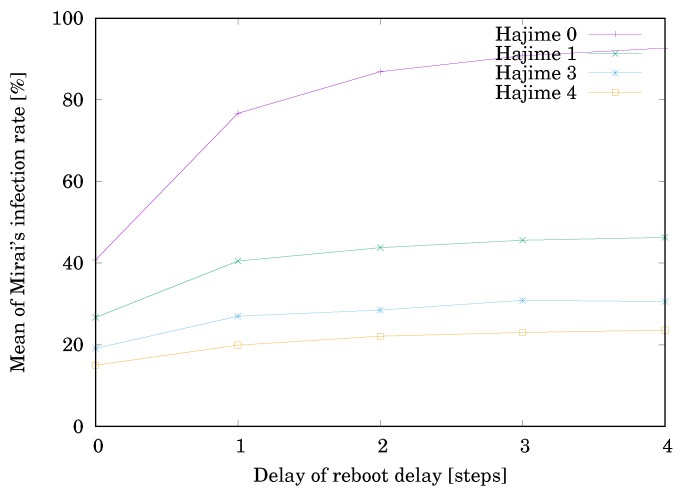
Effect of Hajime against Mirai.

Table 1 shows the simulation result. Each value is the mean of $R_{Mirai}$ for 10000 trials. The result is illustrated in Figure 3. The horizontal axis shows the delay time δ until rebooting. The vertical axis shows Mirai’s infection rate $R_{Mirai}$. First, let us see the effect of only reboot, i.e., when ${\#}_{Hajime}^{init}=0$. Rebooting infected devices drastically reduces the value of $R_{Mirai}$ when δ=0, but the effect is rapidly lost with the increase in δ. This is consistent with the fact [20] that, if the devices are not updated on security, they can be reinfected within minutes of the reboot. Next, let us see on the effect of Hajime, i.e., when ${\#}_{Hajime}^{init}\geq 1$. Hajime reduces the value of $R_{Mirai}$ to less than half without depending on δ. $R_{Mirai}$ decreased with the increase in ${\#}_{Hajime}^{init}$, but the reduction rate gradually decreased. The reason is that the network became saturated with Hajime.

## 3. White-Hat Worm

### 3.1. Analysis and Design

The number of IoT devices is exponentially increasing. This fact makes Mirai’s threat more serious. We need to manage their vulnerability against Mirai, but human-wave tactics are unrealistic because of the huge amount. In this paper, we propose a new approach that uses a white-hat worm to fight Mirai.

Hajime actually protects IoT devices from Mirai’s infection. However, a new problem appears here. Those devices became infected by Hajime instead. Is Hajime a white-hat worm? Once Hajime infects an IoT device, it displays a message for warning the user. At present, there is not any DDoS capability in Hajime. However, Hajime can add new capabilities on the fly, which is an unfavorable attribute. In addition, Hajime continues to stay at the infected device even though completing the defense against Mirai. From these reasons, Hajime is said to be gray-hat.

We extend Hajime to become a white-hat worm. The white-hat worm should not stay at the device once the protection completed. To achieve this, we introduce a concept of lifespan. The white-hat worm destructs itself when exhausting the lifespan. We also introduce a concept of secondary infectivity, which is the ability to infect a device infected by Mirai. This enables the white-hat worm to drive out Mirai.

### 3.2. Modeling

**Figure 4:**
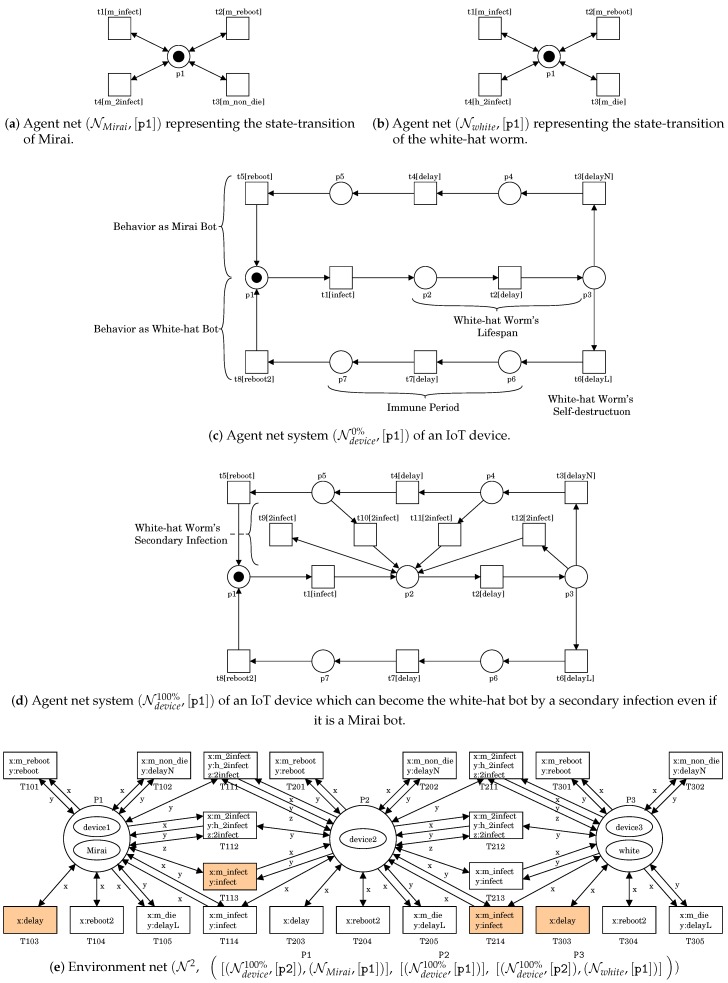
A PN${}^{2}$ system representing a battle between Mirai and the white-hat worm.

To express a battle between Mirai and the white-hat worm, we extend the PN${}^{2}$ model $\left(N^{2},s_{0}\right)$ described in Section 2.2. The extended PN${}^{2}$ model is denoted by $\left(N^{2},s_{0}\right)$ and is shown in Figure 4. The agent net $N_{white}$ of Figure 4b represents the state-transition of the white-hat worm. It is an extension of $N_{Hajime}$ of Figure 1b. Transition t3 labeled as m_die represents a self-destruction action. Transition t4 labeled as h_2infect represents a secondary infection action.

The agent net $N_{Mirai}$ of Figure 4a represents the state-transition of Mirai. It is the same structure as $N_{white}$. However, transition t3 is labeled as m_non_die and represents an action of doing nothing unlike the white worm’s self-destruction action. Transition t4 is labeled as m_2infect and represents a secondary infection action by the white-hat worm.

The agent net $N_{device}^{0\%}$ of Figure 4c represents the state-transition of an IoT device. It is an extension of $N_{device}$ of Figure 1c. For symbol $N_{device}^{0\%}$, its superscript “0%” indicates the possibility of the white-hat worm’s secondary infection. That is, this white-hat worm does not have any secondary infectivity against Mirai. $N_{device}^{0\%}$ has a branch structure at place p3. Which transition t3 or t6 to fire is decided by dynamic binding. If this device is infected by Mirai, t3 would fire. The upper cycle p1t1p2t2p3t3p4t4p5t5p1 represents the behavior as a Mirai bot. If this device is infected by the white-hat worm, t6 would fire. The lower cycle p1t1p2t2p3t6p6t7p7t8p1 represents the behavior as a white-hat bot. Note that each cycle corresponds to $N_{device}$ of Figure 1c. In this example, the white-hat worm’s lifespan is assumed to be one step, of which the delay is represented by transition t2. t6 labeled as delayL represents the white-hat worm’s self-destruction action. Note that the remaining time until reboot means the period of immunity provided by the white-hat worm.

Figure 4d shows the agent net $N_{device}^{100\%}$, where the possibility of this white-hat worm’s secondary infection is 100%. That is, the white-hat worm can always infect the device infected by Mirai. In $\left(N_{device}^{100\%},\left[p1\right]\right)$, the four states [p2],[p3],[p4] and [p5] mean that the device is a Mirai bot. Transitions t9, t10, t11, and t12 respectively represent the white-hat worm’s secondary infection actions. The firing of one transition results in the state [p2] in which the white-hat worm infected the device instead of Mirai. Since those four transitions one-to-one correspond to all of the four states, the white-hat worm’s secondary infection becomes 100%. We can specify any possibility of the white-hat worm’s secondary infection by the presence of those transitions.

The environment net $N^{2}$ of Figure 4e represents the same IoT network as Figure 1d. However, place P3 possesses a token representing the white-hat worm instead of Hajime. This means that the white-hat worm infects device3. The state of Figure 4e is written as follows:

$$\begin{array}{c} s_{0}=\left(\overset{P1}{[\left(N_{device}^{100\%},\left[p2\right]\right),\left(N_{Mirai},\left[p1\right]\right)],}\;\overset{P2}{[\left(N_{device}^{100\%},\left[p1\right]\right)],}\;\overset{P3}{\left[\left(N_{device}^{100\%},\left[p2\right]\right),\left(N_{white},\left[p1\right]\right)\right]}\right). \end{array}$$

### 3.3. Simulation

**Figure 5:**
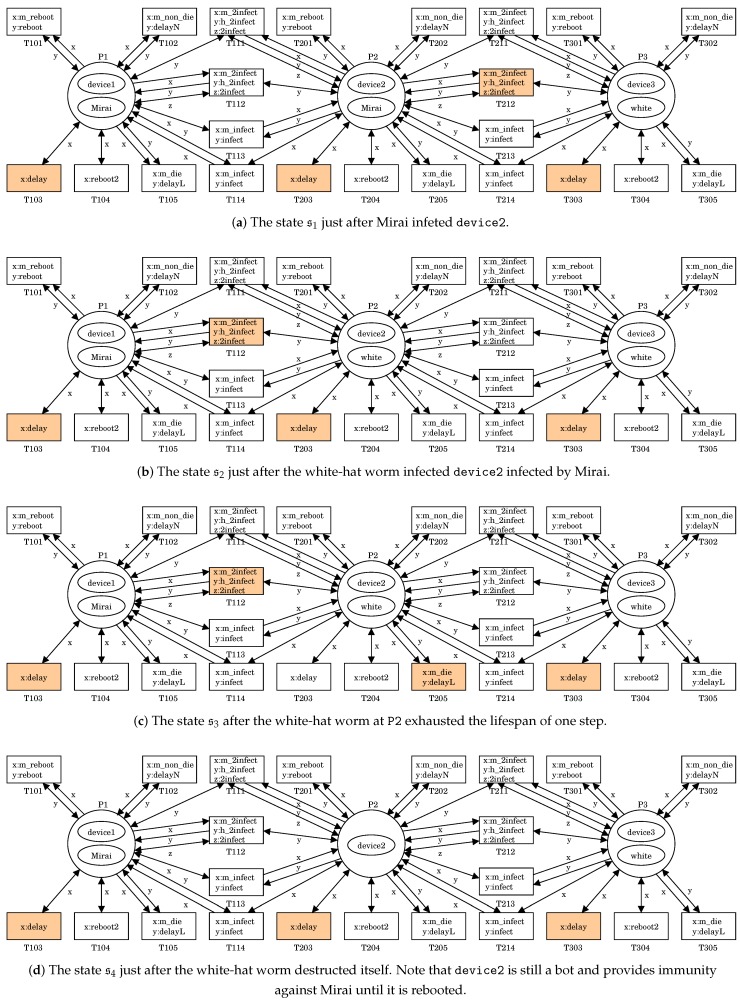
An execution of $\left(N^{2},s_{0}\right)$.

We can simulate the battle between Mirai and the white-hat worm by using the PN${}^{2}$ model proposed in Section 3.2. Figure 5 shows an execution of $\left(N^{2},s_{0}\right)$. Note that $\left(N^{2},s_{0}\right)$ is shown in Figure 4e. In $s_{0}$, there are four firable transitions T103, T113, T214, and T303. Let us fire T113. It means that Mirai infects device2. This results in a new state $s_{1}=$

$$\begin{array}{c} \left(\overset{P1}{[\left(N_{device}^{100\%},\left[p2\right]\right),\left(N_{Mirai},\left[p1\right]\right)],}\;\overset{P2}{[\left(N_{device}^{100\%},\left[p2\right]\right),\left(N_{Mirai},\left[p1\right]\right)],}\;\overset{P3}{\left[\left(N_{device}^{100\%},\left[p2\right]\right),\left(N_{white},\left[p1\right]\right)\right]}\right) \end{array}$$

shown in Figure 5a.

In $s_{1}$, there are four firable transitions T103, T203, T212, and T303 because

- For T103, T203, or T303, x:delay can be bounded with t2 in $\left(N_{device}^{100\%},\left[p2\right]\right)$.
- For T212, x:m_2infect, y:h_2infect and z:2infect can be respectively bounded with t4 in $\left(N_{Mirai},\left[p1\right]\right)$ at P2, t4 in $\left(N_{white},\left[p1\right]\right)$ at P3 and t9 in $\left(N_{device}^{100\%},\left[p2\right]\right)$ at P2.

Let us fire T212. It means the white-hat worm’s secondary infection for device2 infected by Mirai, i.e., the white-hat worm at P3 removes Mirai from P2 and produces a copy of itself into P2, and the copy infects device2. This results in a new state $s_{2}=$

$$\begin{array}{c} \left(\overset{P1}{[\left(N_{device}^{100\%},\left[p2\right]\right),\left(N_{Mirai},\left[p1\right]\right)],}\;\overset{P2}{[\left(N_{device}^{100\%},\left[p2\right]\right),\left(N_{white},\left[p1\right]\right)],}\;\overset{P3}{\left[\left(N_{device}^{100\%},\left[p2\right]\right),\left(N_{white},\left[p1\right]\right)\right]}\right) \end{array}$$

shown in Figure 5b.

In $s_{2}$, there are four firable transitions T103, T112, T203, and T303 because

- For T103, T203, or T303, x:delay can be bounded with t2 in $\left(N_{device}^{100\%},\left[p2\right]\right)$.
- For T112, x:m_2infect, y:h_2infect and z:2infect can be respectively bounded with t4 in $\left(N_{Mirai},\left[p1\right]\right)$ at P1, t4 in $\left(N_{white},\left[p1\right]\right)$ at P2 and t9 in $\left(N_{device}^{100\%},\left[p2\right]\right)$ at P1.

Let us fire T203. It means that the white-hat worm exhausts the lifespan of one step. This results in a new state $s_{3}=$

$$\begin{array}{c} \left(\overset{P1}{[\left(N_{device}^{100\%},\left[p2\right]\right),\left(N_{Mirai},\left[p1\right]\right)],}\;\overset{P2}{[\left(N_{device}^{100\%},\left[p3\right]\right),\left(N_{white},\left[p1\right]\right)],}\;\overset{P3}{\left[\left(N_{device}^{100\%},\left[p2\right]\right),\left(N_{white},\left[p1\right]\right)\right]}\right) \end{array}$$

shown in Figure 5c.

In $s_{3}$, there are four firable transitions T103, T112, T205, and T303. For T205, x:m_die and y:delayL can be respectively bounded with t3 in $\left(N_{white},\left[p1\right]\right)$ at P2 and t6 in $\left(N_{device}^{100\%},\left[p2\right]\right)$ at P2. Let us fire T205. It means that the white-hat worm destructs itself. This results in a new state $s_{4}=$

$$\begin{array}{c} \left(\overset{P1}{[\left(N_{device}^{100\%},\left[p2\right]\right),\left(N_{Mirai},\left[p1\right]\right)],}\;\overset{P2}{[\left(N_{device}^{100\%},\left[p6\right]\right)],}\;\overset{P3}{\left[\left(N_{device}^{100\%},\left[p2\right]\right),\left(N_{white},\left[p1\right]\right)\right]}\right) \end{array}$$

shown in Figure 5d. Note that device2 is still a bot and provides immunity against Mirai until it is rebooted.

## 4. Simulation Evaluation

**Figure 6:**
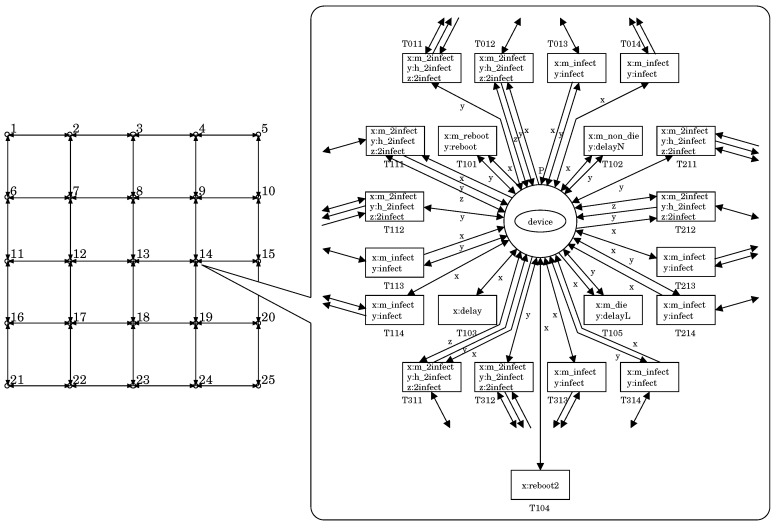
Illustration of the PN${}^{2}$ model used in the experiment.

We performed an experiment to evaluate the effect of the white-hat worm. In this experiment, we used the PN${}^{2}$ model representing a lattice-structured network composed of 25 (=5×5) nodes, i.e., ${\#}_{device}$ = 25. Each node has one device. Figure 6 illustrates the model.

Let us first focus on the white-hat worm’s lifespan. We measured Mirai’s infection rate $R_{Mirai}$ given by Equation (1) and the white-hat worm’s infection rate $R_{white}$ after 1000 steps. $R_{white}$ is given by

(2) $$R_{white}=\frac{{\#}_{white}}{{\#}_{device}},$$

where ${\#}_{white}$ is the number of devices infected by the white-hat worm. The parameters are as follows:

- The delay time δ until rebooting = 7 or 11 steps,
- The initial number ${\#}_{Mirai}^{init}$ of devices infected by Mirai = 12,
- The initial number ${\#}_{white}^{init}$ of devices infected by the white-hat worm = 5,
- The white-hat worm’s lifespan *ℓ* = 1, 3, or 5 steps,
- The white-hat worm’s secondary infection possibility ρ = 100%.

**Figure 7:**
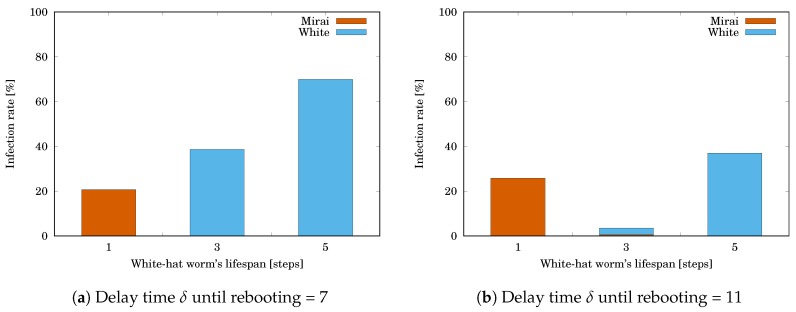
Effect of the white-hat worm’s lifespan *ℓ* on Mirai’s and the white-hat worm’s infection rates.

The simulation results are shown in Table 2. Table 2a shows Mirai’s infection rate $R_{Mirai}$ and the white-hat worm’s infection rate $R_{white}$ when the delay time δ until rebooting =7. Table 2b shows $R_{Mirai}$ and $R_{white}$ when δ=11. Each value is the mean of $R_{Mirai}$ or $R_{white}$ for 10,000 trials. The grayed cell means that the value is getting worse than the initial one. Figure 7a,b respectively illustrate the tables. The horizontal axis shows the white-hat worm’s lifespan *ℓ*. The vertical axis shows $R_{Mirai}$ and $R_{white}$. In both cases, $R_{Mirai}$ was rapidly decreasing with increasing *ℓ*. In contrast, $R_{white}$ started at zero when ℓ=1 and increased with increasing *ℓ*. This means that, if the lifespan is short, it successfully reduces the white-hat worm’s remaining.

Next, let us focus on the white-hat worm’s secondary infectivity. We measured $R_{Mirai}$ and $R_{white}$ after 1000 steps by varying the following parameters.

- The white-hat worm’s secondary infection possibility ρ = 0, 25, 50, 75, or 100%

The other parameters are the same as the previous simulation.

**Figure 8:**
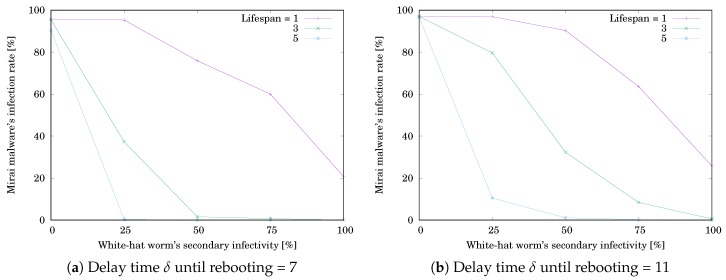
Effect of the white-hat worm’s secondary infectivity ρ on Mirai’s infection rate $R_{Mirai}$ when ${\#}_{device}=25$.

**Figure 9:**
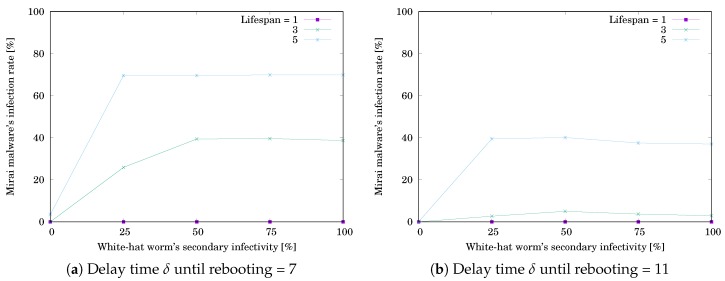
Effect of the white-hat worm’s secondary infectivity ρ on the worm’s infection rate $R_{white}$ when ${\#}_{device}=25$.

The simulation results are shown in Table 3 and Table 4. Table 3a,b respectively show $R_{Mirai}$ when δ=7 and 11. Table 4a,b respectively show $R_{white}$ when δ=7 and 11. Each value is the mean of $R_{Mirai}$ or $R_{white}$ for 10,000 trials. The grayed cell means that the value is getting worse than the initial one. Figure 8a,b respectively illustrate $R_{Mirai}$ when δ=7 and 11. The horizontal axis shows the white-hat worm’s secondary infection possibility ρ. The vertical axis shows $R_{Mirai}$. $R_{Mirai}$ was decreasing with increasing ρ. Note that the decreasing rate depends on the lifespan *ℓ*. Figure 9a,b respectively illustrate $R_{white}$ when δ=7 and 11. The horizontal axis shows ρ. The vertical axis shows $R_{white}$. $R_{white}$ was increasing with increasing ρ and reached a ceiling. Note that the increasing rate depends on *ℓ*. The result means that, if ρ is low, the white-hat worm’s effect depends on *ℓ*. If ρ is high, the worm is effective without depending on *ℓ*.

The effect of the white-hat worm would be influenced by the other factors, e.g., the number of nodes, the connectivity of the nodes, and so on. To investigate how much the number of nodes affects the effect, we performed another experiment. In this experiment, we used the PN${}^{2}$ model representing a larger lattice-structured network. The network consists of 36 (=6×6) nodes, i.e., ${\#}_{device}$ = 36. We measured $R_{Mirai}$ and $R_{white}$ after 1000 steps. The parameters are as follows:

- The initial number ${\#}_{Mirai}^{init}$ of devices infected by Mirai = 18,
- The initial number ${\#}_{white}^{init}$ of devices infected by the white-hat worm = 7.

The other parameters are the same as the previous simulation.

**Figure 10:**
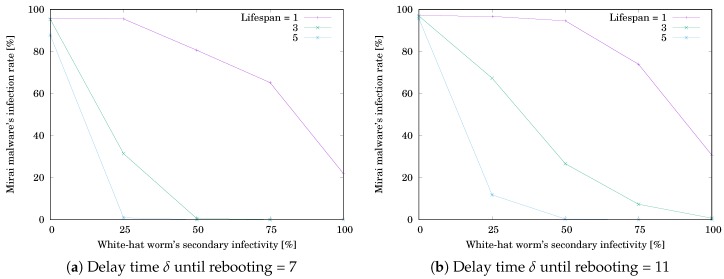
Effect of the white-hat worm’s secondary infectivity ρ on Mirai’s infection rate $R_{Mirai}$ when ${\#}_{device}=36$.

**Figure 11:**
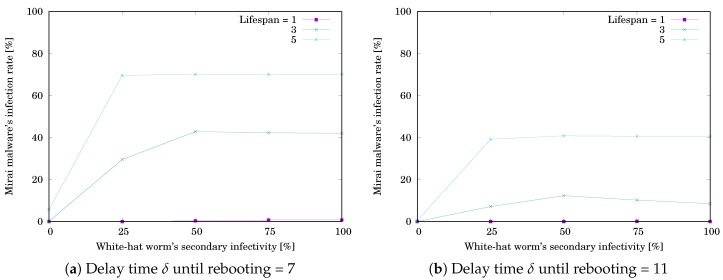
Effect of the white-hat worm’s secondary infectivity ρ on the worm’s infection rate $R_{white}$ when ${\#}_{device}=36$.

The simulation results are shown in Table 5 and Table 6. Table 5a,b respectively show $R_{Mirai}$ when δ=7 and 11. Table 6a,b respectively show $R_{white}$ when δ=7 and 11. Each value is the mean of $R_{Mirai}$ or $R_{white}$ for 1000 trials. The grayed cell means that the value is getting worse than the initial one. Figure 10 and Figure 11 respectively illustrate Table 5 and Table 6. The horizontal axis shows the white-hat worm’s secondary infection possibility ρ. The vertical axis shows $R_{Mirai}$ or $R_{white}$. $R_{Mirai}$ was decreasing with increasing ρ, while $R_{white}$ was increasing with increasing ρ and reached a ceiling. However, the changing rates depend on *ℓ*. Comparing the results for ${\#}_{device}=25$ and 36, we see that the trend is similar. We can say that secondary infectivity and lifespan are more important factors than the number of nodes.

## 5. Conclusions

In this paper, we proposed a new approach that uses a white-hat worm to fight malware. We designed the white-hat worm by introducing the concept of lifespan and secondary infectivity to Hajime. The white-hat worm destructs itself when exhausting the lifespan. In addition, it can drive out Mirai from the infected device. We expressed the white-hat worm with PN${}^{2}$ and performed the simulation for evaluating the effect of the white-hat worm against Mirai. The result of the simulation evaluation shows that (i) the lifespan successfully reduces the white-hat worm’s remaining if short; (ii) if the worm has low secondary infectivity, its effect depends on the lifespan; and (iii) if the worm has high secondary infectivity, it is effective without depending on the lifespan.

In future work, we are going to work up the proposed white-hat worm into a new kind of cybersecurity systems, named *Botnet Defense System (BDS)* [21], which defends a network system against malicious botnets.

## Figures and Tables

**Table 1:** Effect of Hajime against Mirai.

The Initial Number	The Delay Time δ until Rebooting				
${\#}_{Hajime}^{init}$ of Hajime	0	1	2	3	4
0	40.8%	76.7%	86.9%	90.8%	92.7%
1	26.7%	40.5%	43.8%	45.6%	46.3%
2	19.1%	27.0%	28.5%	30.9%	30.6%
3	15.0%	19.9%	22.1%	23.0%	23.6%

**Table 2:** Effect of the white-hat worm’s lifespan *ℓ* on Mirai’s and the white-hat worm’s infection rates. (a) Delay time δ until rebooting = 7; (b) Delay time δ until rebooting = 11.

(a)		
**Lifespan *ℓ***	**$R_{\mathrm{Mirai}}$**	**$R_{\mathrm{white}}$**
1	20.7%	0.0%
3	0.0%	38.7%
5	0.0%	69.9%
(**b**)		
**Lifespan *ℓ***	**$R_{\mathrm{Mirai}}$**	**$R_{\mathrm{white}}$**
1	25.8%	0.0%
3	0.6%	2.9%
5	0.0%	37.0%

**Table 3:** Effect of the white-hat worm’s secondary infectivity ρ on Mirai’s infection rate $R_{Mirai}$ when ${\#}_{device}=25$. (a) Delay time δ until rebooting = 7; (b) Delay time δ until rebooting = 11.

(a)					
**Lifespan *ℓ***	**White-Hat Worm’s Secondary Infectivity ρ**				
	**0%**	**25%**	**50%**	**75%**	**100%**
1	95.6%	95.3%	75.9%	60.0%	20.7%
3	95.5%	37.3%	1.6%	0.7%	0.0%
5	90.4%	0.3%	0.0%	0.0%	0.0%
(**b**)					
**Lifespan *ℓ***	**White-Hat Worm’s Secondary Infectivity ρ**				
	**0%**	**25%**	**50%**	**75%**	**100%**
1	97.0%	96.9%	90.3%	63.6%	25.8%
3	97.0%	79.6%	32.3%	8.4%	0.6%
5	96.7%	10.5%	1.1%	0.2%	0.0%

**Table 4:** Effect of the white-hat worm’s secondary infectivity ρ on the worm’s infection rate $R_{white}$ when ${\#}_{device}=25$. (a) Delay time δ until rebooting = 7; (b) Delay time δ until rebooting = 11.

(a)					
**Lifespan *ℓ***	**White-Hat Worm’s Secondary Infectivity** ρ				
	**0%**	**25%**	**50%**	**75%**	**100%**
1	0.0%	0.0%	0.0%	0.0%	0.0%
3	0.0%	25.9%	39.4%	39.6%	38.7%
5	3.5%	69.6%	69.6%	69.9%	69.9%
(**b**)					
**Lifespan *ℓ***	**White-Hat Worm’s Secondary Infectivity ρ**				
	**0%**	**25%**	**50%**	**75%**	**100%**
1	0.0%	0.0%	0.0%	0.0%	0.0%
3	0.0%	2.7%	5.0%	3.7%	2.9%
5	0.1%	39.5%	40.1%	37.5%	37.0%

**Table 5:** Effect of the white-hat worm’s secondary infectivity ρ on Mirai’s infection rate $R_{Mirai}$ when ${\#}_{device}=36$. (a) Delay time δ until rebooting = 7; (b) Delay time δ until rebooting = 11.

(a)					
**Lifespan *ℓ***	**White-Hat Worm’s Secondary Infectivity ρ**				
	**0%**	**25%**	**50%**	**75%**	**100%**
1	95.7%	95.5%	80.6%	65.2%	22.0%
3	95.3%	31.4%	0.5%	0.1%	0.0%
5	87.7%	0.9%	0.0%	0.0%	0.0%
(**b**)					
**Lifespan *ℓ***	**White-Hat Worm’s Secondary Infectivity ρ**				
	**0%**	**25%**	**50%**	**75%**	**100%**
1	97.1%	96.7%	94.6%	73.9%	30.7%
3	96.9%	67.3%	26.6%	7.3%	0.7%
5	95.7%	11.8%	0.3%	0.0%	0.0%

**Table 6:** Effect of the white-hat worm’s secondary infectivity ρ on the worm’s infection rate $R_{white}$ when ${\#}_{device}=36$. (a) Delay time δ until rebooting = 7; (b) Delay time δ until rebooting = 11.

(a)					
**Lifespan *ℓ***	**White-Hat Worm’s Secondary Infectivity ρ**				
	**0%**	**25%**	**50%**	**75%**	**100%**
1	0.0%	0.0%	0.3%	0.7%	0.7%
3	0.1%	29.5%	42.8%	42.3%	42.0%
5	5.7%	69.6%	70.1%	70.1%	70.1%
(**b**)					
**Lifespan *ℓ***	**White-Hat Worm’s Secondary Infectivity ρ**				
	**0%**	**25%**	**50%**	**75%**	**100%**
1	0.0%	7.1%	12.3%	0.1%	0.0%
3	0.0%	0.2%	0.4%	10.2%	8.5%
5	0.4%	39.2%	40.8%	40.6%	40.6%
